# A Pediatric Case of Hepatocellular Malignant Neoplasm, Not Otherwise Specified (HCN‐NOS) With Poor Prognosis Post‐Tumor Resection and Postoperative Chemotherapy

**DOI:** 10.1002/cnr2.70378

**Published:** 2025-10-23

**Authors:** Bin Yamaoka, Reina Hoshi, Kako Ono, Takayuki Hirano, Yosuke Watanabe, Shumpei Goto, Takashi Hosokawa, Shuichiro Uehara

**Affiliations:** ^1^ Department of Pediatric Surgery Nihon University School of Medicine Tokyo Japan

**Keywords:** children, hepatoblastoma, hepatocellular carcinoma, hepatocellular malignant neoplasm

## Abstract

**Introduction:**

Hepatocellular malignant neoplasm, not otherwise specified (HCN‐NOS) is a newly identified liver tumor with features of mixed hepatoblastoma (HB) and hepatocellular carcinoma (HCC). Although its prognosis and treatment strategies are not well defined, some reports suggested that complete resection with HB‐targeting chemotherapy may yield favorable outcomes.

**Case Presentation:**

We present herein a pediatric case of HCN‐NOS in which the tumor was fully resected followed by postoperative chemotherapy. Unfortunately, the patient subsequently died.

**Conclusion:**

This case highlights the fact that hepatoblastoma protocols may not always be effective in the treatment of HCN‐NOS; therefore, developing treatment strategies tailored to HCN‐NOS is crucial to improve patient prognosis.

**Abbreviations**
5‐FU: fluorouracil
AFP: alpha‐fetoprotein
CBDCA: carboplatin
CDDP: cisplatin
CPT‐11: irinotecan
CT: computed tomography
DXR: doxorubicin
GEM: gemcitabine
HB: hepatoblastoma
HCC: hepatocellular carcinoma
HCN‐NOS: hepatocellular malignant neoplasm, not otherwise specified
IF: ifosfamide
L‐OHP: oxaliplatin
MRI: magnetic resonance imaging
PLADO: CDDP + DXR
TP: topotecan
VCR: vincristine
VP‐16: etoposide

## 1 Introduction

Hepatoblastoma (HB) is the most common type of malignant liver tumor in children, accounting for 70%–80% of such cancers [1]. The peak age of onset of HB is between 6 months and 3 years, with only 9% of cases occurring past 4 years of age [2]. The second most common malignant liver tumor in children is hepatocellular carcinoma (HCC), which is more common in older children [1, 3]. The prognoses of these conditions differ significantly, with a 5‐year survival rate of approximately 70% for HB compared to only 30% for HCC [4].

Hepatocellular malignant neoplasm, not otherwise specified (HCN‐NOS), with characteristics of both HB and HCC, has recently been reported as a new disease concept in pediatric malignant liver tumors. What makes our case noteworthy is the adolescent onset with HB‐predominant mixed histology, R0 resection at presentation (PRETEXT I), and very early distant relapse despite adjuvant HB‐protocol chemotherapy (PLADO)—a pattern that underscores the limitations of extrapolating HB regimens to HCN‐NOS. Recent series also suggest that HCN‐NOS may harbor higher‐risk clinicopathologic and genomic features than classic HB [5], lending biological plausibility to the aggressive course observed here.

## 2 Case Presentation

**FIGURE 1:**
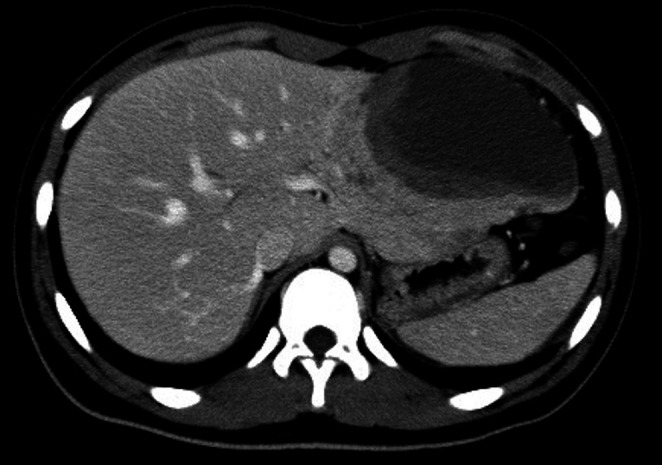
A contrast‐enhanced CT of the abdomen revealed a low‐attenuation mass (12 × 8 × 6 cm) in the left lateral lobe of the liver with poor enhancement that was observed in the lateral segment of the left hepatic lobe.

A 15‐year‐old boy presented to Nihon University Itabashi Hospital, Tokyo, Japan, in August 2016 with epigastric pain which started the day prior. His abdomen was flat and soft, with tenderness in the area of the pericardial fossa, although no palpable masses were appreciated. Contrast‐enhanced computed tomography (CT) of the abdomen revealed a 12 × 8 × 6 cm tumor in the left lateral lobe of the liver (Figure 1), with no distant metastases observed. To evaluate hepatocellular carcinoma in the differential diagnosis, magnetic resonance imaging (MRI) was performed. It revealed a well‐defined mass with low signal intensity without contrast effects on contrast‐enhanced T1‐weighted images and an isointense signal on T2‐weighted images. On gadoxetic acid (Gd‐EOB‐DTPA)–enhanced MRI, the lesion was hypointense on the hepatobiliary phase relative to the surrounding liver parenchyma. A laboratory examination of the blood revealed an alpha‐fetoprotein (AFP) of 5353 (institutional adolescent reference ≤ 20 ng/mL) ng/mL and protein induced by vitamin K absence or antagonist II was 23 mAU/mL (reference ≤ 40 mAU/mL); additionally, there were elevations in the white blood cell count (13 100/μL), total bilirubin (1.75 mg/dL), aspartate aminotransferase (AST, 392 U/L), alanine aminotransferase (ALT, 502 U/L), lactate dehydrogenase (LDH, 463 U/L), alkaline phosphatase (ALP, 463 U/L), and C‐reactive protein (CRP, 8.1 mg/dL). Due to his age, HCC (cT2N0M0: stage II) was suspected. The patient underwent left lateral segmentectomy without prior neoadjuvant chemotherapy.

**FIGURE 2:**
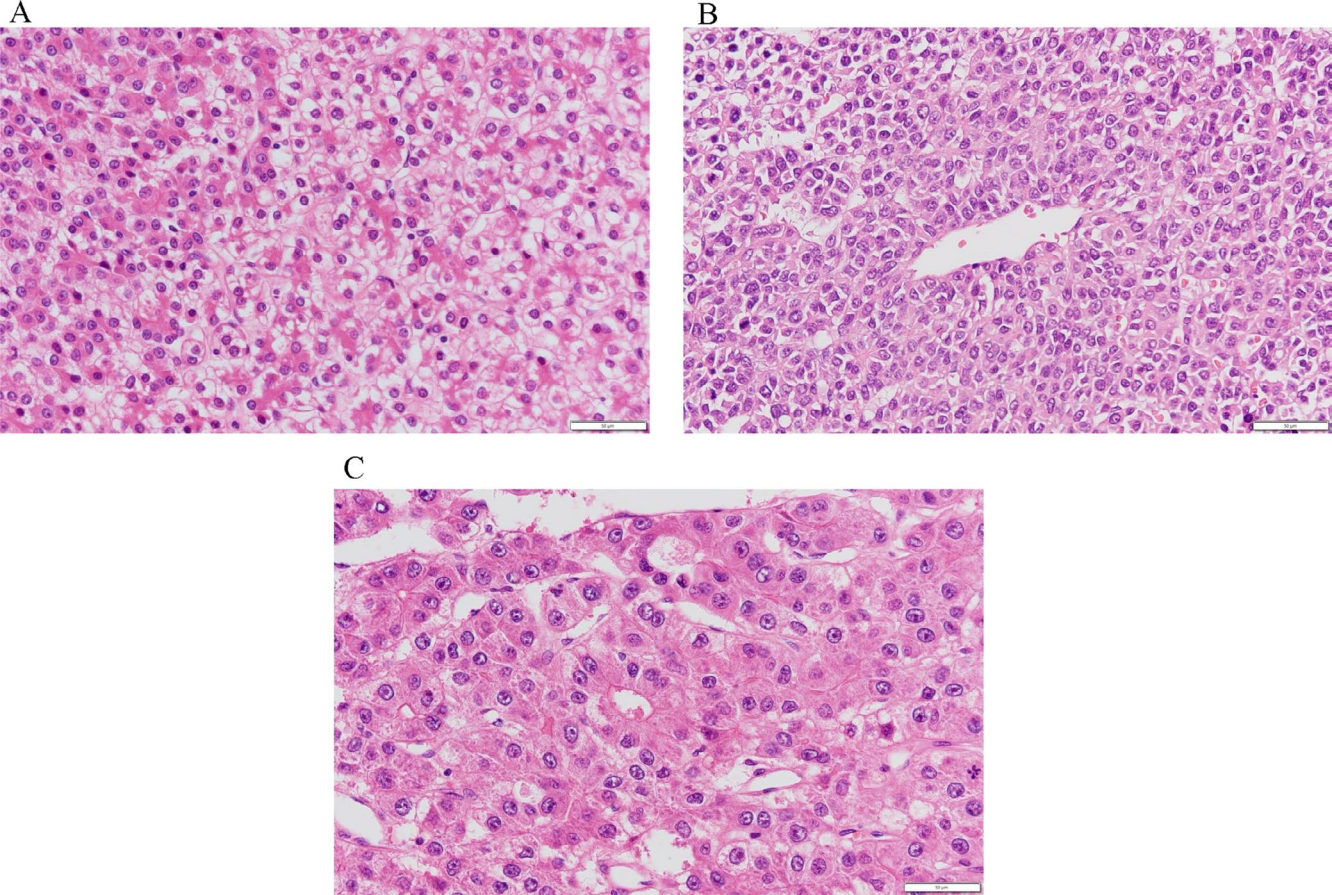
Histopathological examination (hematoxylin–eosin staining, 40×). (A) Fetal HB‐like area with slightly larger tumor cells and low nuclear‐to‐cytoplasmic ratio. (B) Embryonal HB‐like area with small cells and high nuclear‐to‐cytoplasmic ratio. (C) HCC‐like area with eosinophilic cells arranged in a sinusoidal cord‐like pattern.

Histopathological evaluation of the tumor revealed a fetal HB component with slightly larger tumor cells and a low nuclear‐to‐cytoplasmic ratio, an embryonal HB component with smaller cells and a high nuclear‐to‐cytoplasmic ratio, and an HCC component with acidophilic cells showing sinusoidal structures (Figure 2), among which the HB components were dominant. On final pathology, the extent of resection corresponded to a gross total resection with negative microscopic margins. After surgery, we administered three courses of cisplatin (CDDP 80 mg/m^2^, day 1) + doxorubicin (DXR 30 mg/m^2^, day 2–3) (PLADO) according to the guidelines for PRETEXT I HB. The patient's AFP subsequently normalized and he was discharged 4 months after the initial diagnosis.

**FIGURE 3:**
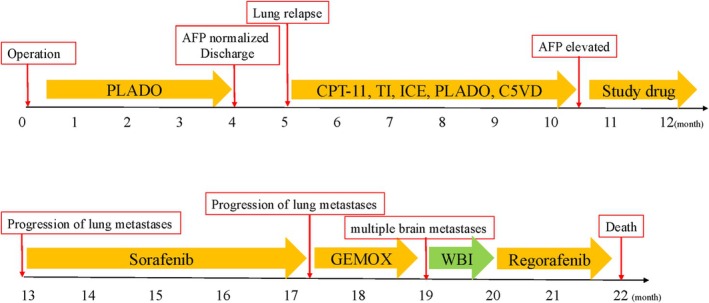
From diagnosis (M0) with operation (left lateral segmentectomy) to outcome at M + 22. M + 1–4: Adjuvant PLADO with AFP normalization and discharge. M + 5: Lung relapse, followed by sequential systemic therapies (CPT‐11; TI; ICE; PLADO; C5VD; study drug; sorafenib; GEMOX) with intermittent lung progression. M + 19: CNS progression → whole‐brain irradiation (WBI) and regorafenib; M + 22: Death. Abbreviations: AFP, alpha‐fetoprotein; PLADO, cisplatin + doxorubicin; CPT‐11, irinotecan; TI, topotecan + ifosfamide; ICE, ifosfamide + carboplatin + etoposide; C5VD, cisplatin + 5‐fluorouracil + vincristine + doxorubicin; GEMOX, gemcitabine + oxaliplatin; WBI, whole‐brain irradiation.

However, five months after the initial diagnosis, a renewed rise in serum AFP prompted evaluation for metastatic relapse, and follow‐up CT demonstrated bilateral lung metastases. A lung biopsy was subsequently performed, which revealed histopathological findings similar to those of the HB component of the primary tumor. No evidence of local recurrence was identified at the primary tumor bed. Although we administered chemotherapy with irinotecan (CPT‐11 100 mg/m^2^), topotecan (TP 0.75 mg/m^2^, days 1‐5) + ifosfamide (IF 1.2 g/m^2^, days 1‐2) (TI), IF (1.8 g/m^2^, days 1‐5) + carboplatin (CBDCA 400mg/m^2^, day 1) + etoposide (VP‐16 100 mg/m^2^, days 1‐5) (ICE), PLADO, and CDDP (90mg/m2, day1)+ fluorouracil (5‐FU 600mg/m2, day2) + vincristine (VCR 1.5mg/m2, day2, 7) + DXR (30mg/m2, day1, 2) (C5VD), the metastases remained. Additionally, the AFP level did not decrease, and actually showed an increasing trend; therefore, a study drug was started. Unfortunately, the number of metastases continued to increase, and sorafenib (800mg/day → 400mg/day → 600mg/day→ 800mg/day) was initiated. However, the metastases further increased, and gemcitabine (GEM 1000 mg/m^2^, day 1, 8) + oxaliplatin (L‐OHP 130 mg/m^2^, day 2) (GEMOX) was introduced. Nineteen months after the initial diagnosis, the patient began to experience convulsions, and an MRI showed multiple brain metastases. Whole brain irradiation (30 Gy) was administered followed by treatment with regorafenib (41mg/m^2^); however, the patient did not respond to this treatment, and ultimately died 22 months after the initial diagnosis (Figure 3).

## 3 Discussion

This case involved a malignant liver tumor with both HB and HCC components, which was classified as HCN‐NOS, a relatively new disease classification system for childhood liver tumors. HCC was initially suspected as the primary diagnosis given the patient's older age at the time of onset, and as no distant metastases were observed upon the initial examination, a systematic hepatectomy was performed to achieve complete tumor resection. Chemotherapy was administered postoperatively based on the pathological findings; however, early relapse with distant metastases occurred shortly after the treatment was completed, leading to rapid disease progression and ultimately death.

Prokurat et al. [6] first described the classification of transitional cell liver tumors as malignant liver tumors with mixed HB and HCC components, which are more prevalent in older children compared to HB. The concept of HCN‐NOS was introduced at the 2014 Children's Oncology Group Liver Tumors Symposium in Los Angeles [7]. The term HCN‐NOS is now widely used to describe these tumors. Sporadic case reports have provided insights into the molecular profile of this disease [2, 5, 6, 8, 9, 10, 11, 12]; however, the number of reported cases remains limited and no standardized treatment strategy has been established. Although HCN‐NOS is biologically aggressive, the prognostic outcomes vary widely, and a consensus on prognosis has yet to be reached. For instance, while Prokurat et al. [6] reported poor prognoses in patients with HCN‐NOS, Seng et al. [9] and Zhou et al. [2] demonstrated that favorable outcomes could be achieved with HB‐targeting chemotherapy and complete tumor resection, including liver transplantation, when necessary.

In this case, however, despite achieving the complete surgical resection of the tumor and the administration of postoperative HB‐targeting chemotherapy, a favorable outcome was not achieved. Tumor biopsies are typically performed prior to initiating treatment in cases of pediatric liver tumors; however, because of the patient's age at the time of presentation, HCC was suspected and a primary tumor resection was performed. Complete tumor resection is the cornerstone of treatment HCC [13], and this approach was deemed appropriate in this case. Furthermore, primary surgical resection is considered a viable initial treatment option in cases of HB classified as PRETEXT I or II [14]. Pathological evaluation of the resected tumor revealed a mixture of HCC and HB components, ensuring that the HB component was not overlooked. Consequently, postoperative HB‐targeting chemotherapy was promptly initiated.

Although previous reports described favorable outcomes with this approach [2, 9], the present patient had a poor outcome. Notably, the tumor was categorized as PRETEXT I, and postoperative chemotherapy was administered based on the protocol for that risk classification. While cisplatin monotherapy and PLADO achieve favorable results in standard‐risk hepatoblastoma [15], the heterogeneous biology of HCN‐NOS may entail different chemosensitivity. Given the aggressive clinical course observed here, it is conceivable that an intensified, high‐risk HB–based regimen might have been more appropriate, although whether earlier intensification would have altered the outcome remains uncertain.

As this case illustrates, HB‐based regimens may yield poor outcomes in some HCN‐NOS even after complete resection; therefore, multicenter case aggregation and prospective studies are needed to define appropriate, disease‐specific treatment strategies.

## Author Contributions

**Bin Yamaoka:** conceptualization, investigation, and writing – original draft. **Rina Hoshi, Kako Ono, Takayuki Hirano, Yosuke Watanabe, Shumpei Goto and Takashi Hosokawa:** validation, writing – review and editing. **Shuichiro Uehara:** conceptualization, writing – review and editing, and supervision. All the authors have read and agreed to the published version of the manuscript.

## Consent

Written informed consent for the publication of case details and use of images was obtained from the patient's parents.

## Conflicts of Interest

The authors declare no conflicts of interest.

## Data Availability

The data that support the findings of this study are available on request from the corresponding author. The data are not publicly available due to privacy or ethical restrictions.

## References

[1] S. J. Cho , “Pediatric Liver Tumors: Updates in Classification,” Surgical Pathology Clinics 13, no. 4 (2020): 601–623, 10.1016/j.path.2020.09.002. 33183723

[2] S. Zhou , R. Venkatramani , S. Gupta , et al., “Hepatocellular Malignant Neoplasm, NOS: A Clinicopathological Study of 11 Cases From a Single Institution,” Histopathology 71, no. 5 (2017): 813–822, 10.1111/his.13297. 28660626 PMC7521842

[3] A. Darbari , K. M. Sabin , C. N. Shapiro , and K. B. Schwarz , “Epidemiology of Primary Hepatic Malignancies in U.S. Children,” Hepatology 38, no. 3 (2003): 560–566, 10.1053/jhep.2003.50375. 12939582

[4] P. Czauderna , D. Lopez‐Terrada , E. Hiyama , B. Häberle , M. H. Malogolowkin , and R. L. Meyers , “Hepatoblastoma State of the Art: Pathology, Genetics, Risk Stratification, and Chemotherapy,” Current Opinion in Pediatrics 26, no. 1 (2014): 19–28, 10.1097/mop.0000000000000046. 24322718

[5] S. Zhou , S. F. Sarabia , D. Estrine , et al., “Comparative Clinicopathologic and Genomic Analysis of Hepatocellular Neoplasm, Not Otherwise Specified, and Hepatoblastoma,” Modern Pathology 37, no. 2 (2024): 100385, 10.1016/j.modpat.2023.100385. 37992967

[6] A. Prokurat , P. Kluge , A. Kościesza , D. Perek , A. Kappeler , and A. Zimmermann , “Transitional Liver Cell Tumors (TLCT) in Older Children and Adolescents: A Novel Group of Aggressive Hepatic Tumors Expressing Beta‐Catenin,” Medical and Pediatric Oncology 39, no. 5 (2002): 510–518, 10.1002/mpo.10177. 12228909

[7] D. López‐Terrada , R. Alaggio , M. T. de Dávila , et al., “Towards an International Pediatric Liver Tumor Consensus Classification: Proceedings of the Los Angeles COG Liver Tumors Symposium,” Modern Pathology 27, no. 3 (2014): 472–491, 10.1038/modpathol.2013.80. 24008558

[8] H. N. Ozcan , B. Oguz , T. Salim , B. Talim , and M. Haliloglu , “A Rare Malignant Hepatic Tumor of Childhood: Transitional Liver Cell Tumor Revisited,” Journal of the Belgian Society of Radiology 98, no. 2 (2015): 79–81, 10.5334/jbr-btr.770. 30394434

[9] M. S. Seng , B. Berry , J. Karpelowsky , et al., “Successful Treatment of a Metastatic Hepatocellular Malignant Neoplasm, Not Otherwise Specified With Chemotherapy and Liver Transplantation,” Pediatric Blood & Cancer 66, no. 4 (2019): e27603, 10.1002/pbc.27603. 30609257

[10] S. Postovsky , R. Elhasid , G. B. Otte , O. Ben Itzhak , D. Gaitini , and M. W. Ben Arush , “Late Recurrence of Combined Hepatocellular Carcinoma and Hepatoblastoma in a Child: Case Report and Review of the Literature,” European Journal of Pediatric Surgery 11, no. 1 (2001): 61–65, 10.1055/s-2001-12197. 11370988

[11] P. Sumazin , T. L. Peters , S. F. Sarabia , et al., “Hepatoblastomas With Carcinoma Features Represent a Biological Spectrum of Aggressive Neoplasms in Children and Young Adults,” Journal of Hepatology 77, no. 4 (2022): 1026–1037, 10.1016/j.jhep.2022.04.035. 35577029 PMC9524481

[12] Y. Chen Wongworawat , S. F. Sarabia , M. Urbicain , et al., “Molecular Profiling of a Hepatocellular Neoplasm Not Otherwise Specified (HCN‐NOS) Demonstrates Distinct Molecular Features in Hepatoblastoma and HCC‐Like Components,” Pediatric and Developmental Pathology 27, no. 2 (2024): 169–175, 10.1177/10935266231204788. 37903123 PMC11015706

[13] K. J. Riehle , S. A. Vasudevan , A. Bondoc , et al., “Surgical Management of Liver Tumors,” Pediatric Blood & Cancer Jul 2 72 (2024): e31155, 10.1002/pbc.31155. 38953150

[14] R. Meyers , E. Hiyama , P. Czauderna , and G. M. Tiao , “Liver Tumors in Pediatric Patients,” Surgical Oncology Clinics of North America 30, no. 2 (2021): 253–274, 10.1016/j.soc.2020.11.006. 33706899

[15] G. Perilongo , R. Maibach , E. Shafford , et al., “Cisplatin Versus Cisplatin Plus Doxorubicin for Standard‐Risk Hepatoblastoma,” New England Journal of Medicine 361, no. 17 (2009): 1662–1670, 10.1056/NEJMoa0810613. 19846851

